# A Novel Acyl-CoA Beta-Transaminase Characterized from a Metagenome

**DOI:** 10.1371/journal.pone.0022918

**Published:** 2011-08-03

**Authors:** Alain Perret, Christophe Lechaplais, Sabine Tricot, Nadia Perchat, Carine Vergne, Christine Pellé, Karine Bastard, Annett Kreimeyer, David Vallenet, Anne Zaparucha, Jean Weissenbach, Marcel Salanoubat

**Affiliations:** 1 Commissariat à l'Energie Atomique et aux Energies Alternatives, Institut de Génomique, Genoscope, Evry, France; 2 CNRS-UMR 8030, Evry, France; 3 UEVE, Université d'Evry, Evry, France; University Paris Diderot-Paris 7, France

## Abstract

**Background:**

Bacteria are key components in all ecosystems. However, our knowledge of bacterial metabolism is based solely on the study of cultivated organisms which represent just a tiny fraction of microbial diversity. To access new enzymatic reactions and new or alternative pathways, we investigated bacterial metabolism through analyses of uncultivated bacterial consortia.

**Methodology/Principal Findings:**

We applied the gene context approach to assembled sequences of the metagenome of the anaerobic digester of a municipal wastewater treatment plant, and identified a new gene which may participate in an alternative pathway of lysine fermentation.

**Conclusions:**

We characterized a novel, unique aminotransferase that acts exclusively on Coenzyme A (CoA) esters, and proposed a variant route for lysine fermentation. Results suggest that most of the lysine fermenting organisms use this new pathway in the digester. Its presence in organisms representative of two distinct bacterial divisions indicate that it may also be present in other organisms.

## Introduction

Microorganisms are the most abundant and diverse forms of life and are essential in the functioning of all ecosystems. However, despite their importance and ubiquity, only a tiny fraction of them is well understood due to their failure to grow under standard laboratory culture conditions. With this limitation, less than 1% of the total number of microbial species have been isolated in pure cultures [1], [2], [3]. Our knowledge of microbial biodiversity is thus severely impaired by relying solely on cultivated microorganisms, leading to a limited appreciation of functional diversity. Recently the development of metagenomic approaches has opened the window on the richness of uncultured biodiversity [4]. These cultivation-independent techniques have shed light on the functioning of microbial communities and led to major surprises, such as the discovery of a new bacteriorhodopsin in a γ-proteobacterium that has since been found widely represented in different taxa, in diverse oceans [5]; the Photosystem I gene cassettes that were shown to be present in marine virus genomes [6]; the nitrite-driven anaerobic methane oxidation by oxygenic bacteria [7]; the expansion of protein families in these newly-studied ecosystems [8]; or the discovery of multi-kingdom Pfam domains that highlight new biological processes conserved through evolution [9].

Here, we have used the wealth of the metagenomic data extracted from the anaerobic digester of a wastewater treatment plant to explore metabolic capabilities of anaerobic bacteria. Previous studies described the archeal and bacterial molecular diversity of this digester, revealing the occurrence of previously undescribed phylogenetic groups and phylotypes [10]. A quantification of the bacterial diversity was conducted using 16S and 23S rRNA-targeted hybridization. Gram-positive bacteria represented the most abundant phyla (22%), followed by the *Chloroflexi* (20%). *Proteobacteria* and *Bacteroidetes* accounted for 14% each. *Planctomycetes* and *Synergistes* represented less than 2% each while WWE1, a novel phylum, accounted for 12% and could have considerable importance in the community. The genome of “*Candidatus* Cloacamonas acidaminovorans”, an uncultivated representative of this lineage, has been reconstructed *in silico* [11].

In anaerobic digestion, microorganisms break down organic material in the absence of oxygen. Three main groups of microorganisms are involved: fermenting bacteria, organic acid oxidizing bacteria, and methanogenic archaea. In a first step, hydrolytic and fermenting bacteria digest the input materials in order to break down complex and polymeric compounds (carbohydrates, proteins…) and make them available for acidogenic bacteria which convert these sugars and amino acids into organic acids. Then acetogenic bacteria convert them into acetic acid. Finally, methanogens convert these products to methane [12]. Carbon dioxide, hydrogen and ammonia are produced during all the steps of this process. “*Candidatus* Cloacamonas acidaminovorans” is considered as a fermentative bacterium, and is suggested to be a hydrogen-producing syntroph [11].

Our previous research on the lysine fermentation pathway at the genetic level [13] and on the predicted metabolism of “*Candidatus* Cloacamonas acidaminovorans” which is supposed to be a lysine fermenting organism [11], led us to re-analyze this metabolic route. We have experimentally identified a novel enzymatic reaction which is involved in a hitherto unobserved variant route for lysine fermentation in this organism.

## Results and Discussion

### Identification of a candidate gene involved in an alternative lysine fermentation pathway

**Figure 1:**
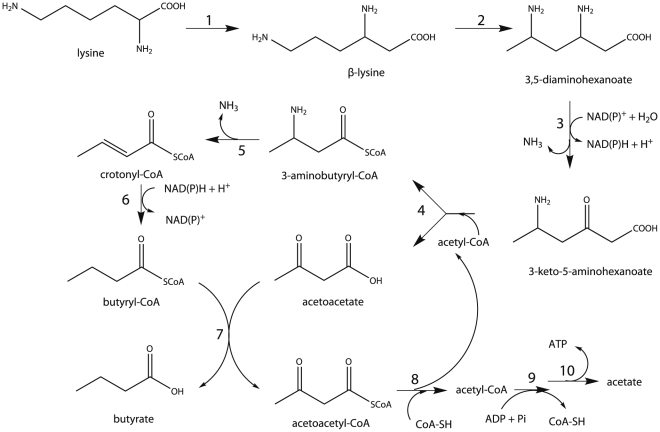
The lysine fermentation pathway. Lysine is converted to β-lysine by lysine-2,3 aminomutase (1) and then to L-*erythro*-3,5-diaminohexanoate by β-lysine-5,6-aminomutase (2). This compound is deaminated and oxidized by a NAD(P)-dependent L-*erythro*-3,5-diaminohexanoate dehydrogenase (3) to yield 3-keto-5-aminohexanoate. It is further converted into 3-aminobutyryl-CoA and acetoacetate in the presence of acetyl-CoA by 3-keto-5-aminohexanoate cleavage enzyme (4). 3-aminobutyryl-CoA is deaminated to crotonyl-CoA by an ammonia lyase (5). Crotonyl-CoA is reduced to butyryl-CoA by butyryl-CoA dehydrogenase (6), which reacts with acetoacetate to form butyrate and acetoacetyl-CoA through the action of acetoacetate:butyrate CoA transferase (7). The latter compound is converted to acetate *via* acetyl-CoA and acetyl phosphate by acetoacetyl-CoA thiolase (8), phosphate acetyltransferase (9), and acetate kinase (10), respectively.

**Figure 2:**
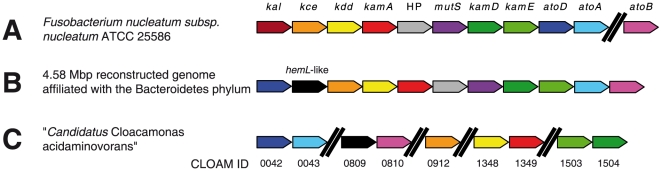
Models of the gene cluster organization for lysine fermentation. (A): *Fusobacterium nucleatum* ATCC 25586; (B): a contig sequence affiliated with the *Bacteroidetes* phylum originating from the metagenome of the anaerobic digester of a wastewater treatment plant; (C): the reconstructed genome of “*Candidatus* Cloacamonas acidaminovorans”, an uncultivated bacterium found in this digester (only the ID number of the CLOAM genes are reported). The symbol “//” means an interruption of >5.5 kb in the cluster. HP indicates a gene encoding a Hypothetical Protein; *mutS* a gene encoding a MutS family protein.

It was demonstrated since the early 1950s that in *Clostridium sticklandii*, lysine was degraded to acetate, butyrate, and ammonia. This catabolic pathway contains ten distinctive reactions (see figure 1). All these genes have been identified [13]. The genome annotation of “*Candidatus* Cloacamonas acidaminovorans” suggested that this bacterium ferments lysine. However, *kal*, responsible for ammonia elimination from 3-aminobutyryl-CoA to produce crotonyl-CoA was absent. One hypothesis was that “*Candidatus* Cloacamonas acidaminovorans” ferments lysine through a variant pathway. We then searched for the genes involved in this alternative route by studying the genomic neighbourhood of the genes involved in lysine fermentation in this bacterium. However, this approach was unfruitful. In this organism, a low percentage of genes are found in groups of co-localized orthologs shared with other species. This led us to study the genomic context of the genes involved in the lysine fermentation pathway in other assembled genomic regions from the anaerobic digester. In particular, a large contig of 50 kbp containing the clustered genes of interest was studied. This contig was later integrated into an *in silico* reconstructed genome of 4.58 Mbp that has been shown to be affiliated with the Bacteroidetes phylum (Fonknechten N. and Le Paslier D., manuscript in preparation). Surprisingly, while the genes involved in lysine fermentation were clustered, *kal* was also missing as observed in “*Candidatus* Cloacamonas acidaminovorans” (figure 2). In these assembled sequences, a gene annotated as “putative glutamate-1-semialdehyde-2,1-aminomutase” (*hemL*) was found at the *kal* locus. A gene also coding for a protein showing strong homology (>65% of sequence identity over the total length of the proteins) to this HemL-like protein was found in “*Candidatus* Cloacamonas acidaminovorans” (CLOAM0809) co-localized with the gene encoding acetoacetyl-CoA thiolase (EC 2.3.1.9; CLOAM0810) (figure 2) that is known to participate to lysine degradation [13].

**Figure 3:**
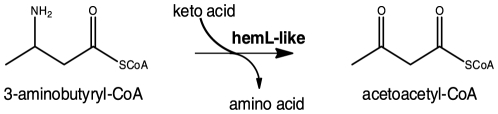
The hypothetical alternative metabolism of 3-aminobutyryl-CoA.

HemL, which converts glutamate-1-semialdehyde to 5-aminolevulinate (EC 5.4.3.8) is an aminomutase/aminotransferase involved in the biosynthesis of porphyrins [14], [15]. However, the *in silico* genome analysis of “*Candidatus* Cloacamonas acidaminovorans” indicated that it does not possess any other gene involved in porphyrin biosynthesis [11]. Together, these data suggested that *hemL*-like was misannotated and was linked in some way to the degradation of lysine, making it a promising candidate for the experimental elucidation of a variant lysine fermentation pathway. Due to the substitution of *kal* in “*Candidatus* Cloacamonas acidaminovorans” and in the reconstructed Bacteroidetes genome, we suspected 3-aminobutyryl-CoA to be metabolized in an unreported manner. We thus focused on HemL-like from “*Candidatus* Cloacamonas acidaminovorans” to analyze how this organism could ferment lysine in the absence of *kal*. Considering the annotated function of HemL-like as an aminomutase/aminotransferase, we hypothesized that it may transfer the amine of 3-aminobutyryl-CoA to an α-keto acid, to directly form acetoacetyl-CoA (Figure 3).

### Purification of the enzymes involved in the alternative lysine fermentation pathway

The recombinant HemL-like, AtoA, and AtoD (the two components of the butyrate-acetoacetate CoA transferase complex) from “*Candidatus cloacamonas acidaminovorans*” were produced in *E. coli* and purified. According to gel filtration experiments, AtoA and AtoD were both recovered as monomers (not shown). Since butyrate-acetoacetate CoA transferase is functional as an α_2_β_2_ heterotetramer [16], [17], an equimolar amount of AtoA and AtoD were mixed and applied onto the gel filtration column to purify the functional complex. The mobility of purified AtoA/AtoD complex on the Superdex 200 gel filtration column (figure S1) indicated that it is a tetramer with an apparent molecular mass of ∼93 kDa (theoretical mass : 101.5 kDa). A sample of purified HemL-like gel filtered under the same conditions yielded an apparent molecular mass of 81 kDa (figure S1), consistent with a dimeric structure (theoretical mass: 94.4 kDa). Since the sequence of the HemL-like possesses an aminotransferase class-III pyridoxal-phosphate (PLP) attachment site (PROSITE motif PS00600), the purified protein was incubated with excess PLP, subjected again to gel filtration, and its spectrum recorded between 250 and 600 nm. The presence of an absorption maximum near 410 nm confirmed that HemL-like is a PLP-dependent protein (figure S2).

### Biochemical and enzymatic characterization of HemL-like

The putative activity of HemL-like was evaluated experimentally by spectrophotometric monitoring at 310 nm of the formation of acetoacetyl-CoA. 3-aminobutyryl-CoA was incubated with HemL-like in the presence of various α-ketoacids (α-ketoglutarate, pyruvate, oxaloacetate, and glyoxylate). Under these conditions, rapid formation of acetoacetyl-CoA was observed in the presence of α-ketoglutarate, and a slower reaction occured with pyruvate. Acetoacetyl-CoA formation was observed in the presence of 3-aminobutyryl-CoA produced both enzymatically and chemically. The enzymatic reaction could also be observed through LC/MS analysis. In the presence of HemL-like, acetoacetyl-CoA was detected in the positive ionization mode at *m/z* 852.1442 (accuracy: 0.7 ppm), and was correlated with a decrease in 3-aminobutyryl-CoA concentration (detected at *m/z* 853.1722 with an accuracy of 3.6 ppm). Its formation was also observed in the presence of both α-ketoglutarate and pyruvate. The kinetic constants of the enzyme were determined spectrophotometrically using (3*S*)-3-aminobutyryl-CoA (Table 1). Therefore, *hemL*-like codes for a 3-aminobutyryl-CoA aminotransferase that we have named *kat*. This novel β-aminotransferase is, to our knowledge, the first that acts on a CoA ester.

**Table 1:** Kinetic parameters of 3-aminobutyryl-CoA transamination.

Substrate	*K*_m_ (M)	*k*_cat_ (s^−1^)	*k*_cat_/*K*_m_ (s^−1^. M^−1^)
3-aminobutyryl-CoA^1^	4.3±1.5 10^−6^	1.80±0.20	4.2 10^5^
α-ketoglutarate^2^	2.9±0.1 10^−3^	1.80±0.10	620
pyruvate^2^	20.1±1.3 10^−3^	0.40±0. 01	20

Values correspond to the average of two replicates.

1 α-ketoglutarate concentration was 50 mM.

2 3-aminobutyryl-CoA concentration was 25 µM.

Its activity in the presence of saturating substrate concentrations was high only in a narrow pH range (7.5–8.5), with an optimum around pH 8.0. The enzyme was inactive below pH 7.0 and exhibited only 20% activity at pH 10 (figure S3a). Kat's activity could not be quantified in HEPES/NaOH nor in Na/K phosphate buffers. At optimal pH, the activity of Kat increased with temperature, up to a maximum at ∼30°C, and sharply decreased with higher temperatures to be undetectable above 33°C (figure S3b). This optimum temperature is similar to that of the digester [18].

**Figure 4:**
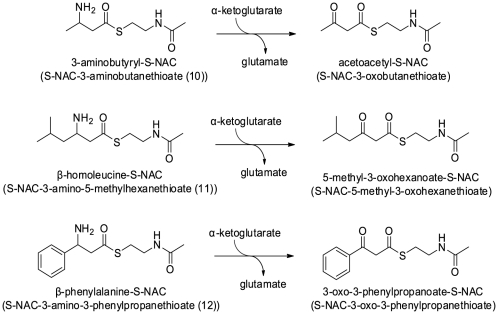
Structure of the different NAC-thioesters and their corresponding products after β-transamination catalyzed by Kat in the presence of α-ketoglutarate.

Kim *et al.* had recently characterized a novel β-aminotransferase from *Mesorhizobium* sp [19], which shows striking properties: its amino acid sequence shows the highest similarity with a glutamate-1-semialdehyde 2,1-aminomutase (53% identity with the enzyme from *Polaromonas* sp. strain JS666, which does not have β-aminocarboxylic transaminase activity). Furthermore, this *Mesorhizobium* β-transaminase shows its highest activity in the presence of α-ketoglutarate as the amino acceptor as well. It has strong activity with β-aminocarboxylic acids, in particular 3-aminobutyrate, which is structurally close to 3-aminobutyryl-CoA. While the enzyme from *Mesorhizobium* sp has only 24% identity with Kat, its properties prompted us to check whether Kat could have a similar activity. Kat was thus assayed for activity in the presence of the best amino donors of the β-transaminase from *Mesorhizobium sp*: 3-aminobutyrate, β-homoleucine and β-phenylalanine. Since none of these compounds could be transformed by Kat, we hypothesized that the CoA moiety is essential for substrate recognition. Previous work has demonstrated that *N*-acetylcysteamine (NAC) thioesters can serve as activated acyl-CoA mimics that are used by the cell [20]. Because the acyl-CoA compounds are difficult to synthesize and to handle due to their high sensitivity to hydrolysis reaction, we chose to use the NAC thioesters. More particularly, the CoA side chain of acetyl-CoA could be substituted by NAC in the reaction catalyzed by the 3-keto-5-aminohexanoate cleavage enzyme (Kce) in the lysine fermentation pathway: in the presence of 3-keto-5-aminohexanoate and acetyl-NAC, the enzyme yielded acetoacetate and 3-aminobutyryl-S-NAC (unpublished results). NAC thioesters of 3-aminobutyrate, β-homoleucine, and β-phenylalanine were thus synthesized and tested as amino donors for Kat. Their structure, along with the corresponding products that would be formed in the presence of α-ketoglutarate and the enzyme are presented in figure 4. Kat was incubated in 10 mM Tris/HCl pH 8.1 in the presence of 20 mM α-ketoglutarate and 50 µM S-NAC-thioester for 2 hours before LC/MS analysis (positive ionization mode). Under these conditions acetoacetyl-S-NAC was detected at *m/z* 204.0682 (Table 2), illustrating that NAC can serve as a CoA mimic for Kat. More interestingly, β-homoleucine-NAC and β-phenylalanine-NAC were also converted to their corresponding oxo-compounds, because 5-methyl-3-oxohexanoate-NAC and 3-oxo-3-phenylpropanoate-NAC were detected at *m/z* 204.0682 and 266.0838, respectively (Table 2). The formation of glutamate was also detected in these three reactions. Additional MS/MS experiments conducted on these three reaction products yielded a common fragment at *m/z* 119.98. This mass is consistent with the protonated form of NAC. Together, these results further confirm the identity of each oxo-metabolite. In conclusion, it appears that the thioester moiety (CoA or its NAC mimic) is actually necessary for substrate recognition, but most of all, Kat is able to transform other β-amino compounds besides 3-aminobutyryl-CoA, including aromatic ones.

**Table 2:** Identification of NAC-thioesters formed by Kat.

		[M+H^+^]^+^			
NAC-thioester	Formula	Theoretical	Observed	Accuracy (ppm)	RT (min)*
acetoacetyl-	C_8_H_13_NO_3_S	204.0689	204.0682	−3.4	6.14
5-methyl-3-oxohexanoate-	C_11_H_19_NO_3_S	246.1158	246.1152	−2.4	8.06
3-oxo-3-phenylpropanoate-	C_11_H_19_NO_3_S	266.0845	266.0838	−2.6	8.02

*RT indicates the retention time of the compound on the chromatography column; [M+H^+^]^+^, protonated molecular ion (positive ionization mode).

### Determination of a variant lysine fermentation pathway

We have demonstrated that *kat* codes for a 3-aminobutyryl-CoA aminotransferase. The concomitant absence of *kal* and presence of *kat* suggests that lysine is metabolized in a different way. In the well-known route, the conversion of 3-aminobutyryl-CoA to acetoacetyl-CoA occurs through three distinct enzymatic steps: 1) ammonia elimination of 3-aminobutyryl-CoA to crotonyl-CoA (EC 4.3.1.14) , 2) reduction of crotonyl-CoA to butyryl-CoA (EC 1.3.99.2) and 3) CoA-SH transfer from butyryl-CoA to acetoacetate (EC 2.8.3.9) [13]. In the proposed alternative route, Kat generates acetoacetyl-CoA, and the AtoA/AtoD complex may yield a second molecule of acetoacetyl-CoA from acetoacetate and acetyl-CoA. Following this scheme, the exergonic reduction of crotonyl-CoA to butyryl-CoA would be suppressed, which could be detrimental to the cell, as this reaction can be coupled to energy conservation *via* electron bifurcation [21], [22]. The genome of “*Candidatus* Cloacamonas acidaminovorans” contains good candidate genes (CLOAM1274, 1482, and 0104) coding for a butyryl-CoA dehydrogenase/electron-transferring-flavoprotein complex (Bcd/EtfAB). However, the formation of crotonyl-CoA from acetoacetyl-CoA or other metabolites remains enigmatic, because no genes coding for 3-hydroxybutyryl-CoA dehydrogenase (EC 1.1.1.157), crotonase (EC 4.2.1.55) or glutaryl-CoA dehydrogenase (EC 1.3.99.7) could be detected. Syntrophic bacteria are able to catalyse the energetically unfavourable formation of crotonyl-CoA from butyryl-CoA using a reverse electron transfer *via* the Rnf system [23]. Since *C. acidaminovorans* is predicted to be a syntroph, one may consider that in this organism the Bcd/Etf complex catalyses the formation of crotonyl-CoA from butyryl-CoA, a reaction coupled to the formation of hydrogen via a formate hydrogen lyase. However sequences of proteins catalysing the oxidative degradation of crotonyl-CoA to acetoacetyl-CoA are not detected in the genome, making this reverse electron transfer rather unlikely. Thus, we can not exclude that the two enzymatic reactions catalysing the formation of crotonyl-CoA from acetoacetyl-CoA are carried out by genes with no homology to those known so far, or that the formation of crotonyl-CoA could be performed through a novel enzymatic sequence.

**Figure 5:**
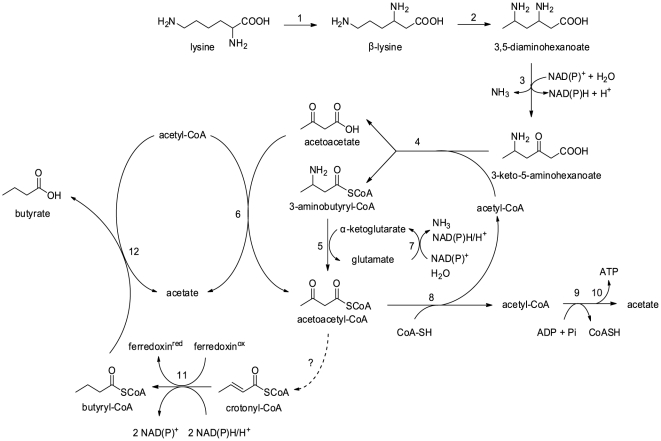
The alternative lysine fermentation pathway. Enzymes involved are L-lysine-2,3-aminomutase (1); β-L-lysine-5,6-aminomutase (2); 3,5-diaminohexanoate dehydrogenase (3); 3-keto-5-aminohexanoate cleavage enzyme (4); 3-aminobutyryl-CoA aminotransferase (Kat; 5); butyrate-acetoacetate CoA transferase (6); glutamate dehydrogenase (7); acetoacetyl-CoA thiolase (8); phosphate acetyltransferase (9); acetate kinase (10); butyryl-CoA dehydrogenase (11); butyrate-acetoacetate CoA transferase (12).

On this basis, a variant lysine fermentation pathway is proposed in figure 5. The divergence with the classical pathway begins in step 5, where acetoacetyl-CoA and glutamate are generated from 3-aminobutyryl-CoA and α-ketoglutarate. The AtoA/AtoD complex can then catalyse the formation of acetoacetyl-CoA and acetate from acetoacetate and acetyl-CoA, yielding a second molecule of acetoacetyl-CoA (step 6). Recycling of α-ketoglutarate may be performed by a glutamate dehydrogenase (step 7). The two acetoacetyl-CoA molecules could be metabolized in two different ways. First, acetoacetyl-CoA can generate ATP and acetate, as described in the classical pathway (Figure 1, steps 8–10). Besides, acetoacetyl-CoA may also be converted to crotonyl-CoA, *via* unidentified enzymatic steps. Afterwards, the butyryl-CoA dehydrogenase complex may carry out the concomitant reduction of crotonyl-CoA and ferredoxin (step 11) [21]. Butyryl-CoA may finally be converted to butyrate and acetyl-CoA in the presence of acetate (step 12). Experimental evidence strengthens our model : the previous characterisation of *kdd* and *kce* in “*Candidatus* Cloacamonas acidaminovorans” [13] validates that L-*erythro*-3,5-diaminohexanoate is actually metabolized to acetoacetate and 3-aminobutyryl-CoA, in this organism. Furthermore in addition to the established function of Kat, the purified AtoA/AtoD complex has been shown to catalyze the formation *in vitro* of acetoacetyl-CoA and acetate from acetoacetate and acetyl-CoA (Table S1).

Thus, cultivable organisms which possess the same gene cluster organization as *F. nucleatum* and “*Candidatus* Cloacamonas acidaminovorans” may ferment lysine to identical products, but through different pathways. This is not totally surprising, as it has been demonstrated that the fermentation of glutamate in *Clostridium tetanomorphum* and *Peptococcus aerogenes* leads to identical products, but proceeds *via* entirely different metabolic pathways [24], [25]. Furthermore, previous studies using tracer experiments have indicated that at least three more or less distinct pathways participate in the process of lysine fermentation in *Clostridia* [26], [27]. The first one corresponds to that described in *F. nucleatum* [13], but another pathway involves a different carbon cleavage in lysine to form butyrate and acetate. Finally, a third pathway involves the formation of butyrate from two acetate or acetyl moieties. Only the first pathway has been established with confidence because there is little data available for the last two.

### Occurrence of kat in genomes and metagenomes

To determine if in the digester lysine is fermented through the canonical or the alternative pathway we searched first in the metagenomic sequences those related to the five genes present in both pathways (i.e *kdd* (coding for the 3,5 diaminohexanoate dehydrogenase), *kce* (coding for the 3-keto-5-aminohexanoate cleavage enzyme, *kamD* (coding for the lysine 5,6-aminomutase α-subunit), and *kamE* (coding for the lysine 5,6 aminomutase β-subunit, *kamA* (coding for the lysine 2,3 aminomutase)) The results are reported in Table 3. *kdd*, *kce*, *kamD* and *kamE* are present between 100 and 200 times. The overabundance of *kamA* could be explained by the similarity between its encoded protein and other aminomutases found in organisms which probably do not ferment lysine. Then we searched for sequences similar to either Kal or Kat. The scarcity of *kal* suggests that a very few organisms present in the digester contained this gene and ferment lysine using the canonical pathway. On the other hand, since *kat* is found with a similar frequency to *kdd*, *kce*, *kamD*, and *kamE*, the alternate pathway may be present in most if not all organisms fermenting lysine in the digester. Kat sequences can be divided into two subgroups composed of almost identical sequences that share ∼65% identity. The first one contains sequences related to WWE1 organisms while in the other one, sequences were affiliated with Bacteroidetes organisms. This indicates that the variant lysine fermentation pathway proceeds in at least two different phyla. A gene (DIG1_60031) from the reconstructed Bacteroidetes genome was cloned and expressed in *E. coli* for protein purification and biochemical characterization. Its enzymatic activity, tested in the presence of 25 µM 3-aminobutyryl-CoA and 30 mM α-ketoglutarate at 28°C in Activity Buffer, showed a similar rate (84%) to the one found for Kat under the same conditions. These data confirm that the variant route can be actually found in distinct phyla.

**Table 3:** Occurrences in the metagenome from the anaerobic digester of a wastewater treatment plant of the genes known to be involved in lysine fermentation.

Gene name	Number of occurrences
*kdd*	124
*kce*	125
*kal*	7
*kamA*	285
*kamD*	184
*kamE*	89
*kat*	159

When Kat is compared to the millions of protein sequences accessible through (1) the Integrated Microbial Genomes interface (http://img.jgi.doe.gov; database : all_img_w_v310.suser), (2) the CAMERA web site [28] (3) large scale gut microbiome survey [29], no clear Kat homolog could be detected. However, when Kat is compared to the NCBI RefSeq collection (http://www.ncbi.nlm.nih.gov/projects/RefSeq/), a protein with 60% identity over the whole length of the sequence was identified (NCBI Reference Sequence: ZP_07200410.1). This protein, annotated as a Class III aminotransferase belongs to the delta proteobacterium NaphS2 [30]. Despite this homology, this cultivable anaerobic marine sulphate-reducing bacterium does not possess the lysine fermentation genes, indicating that the function of its aminotransferase probably differs from that of Kat.

**Figure 6:**
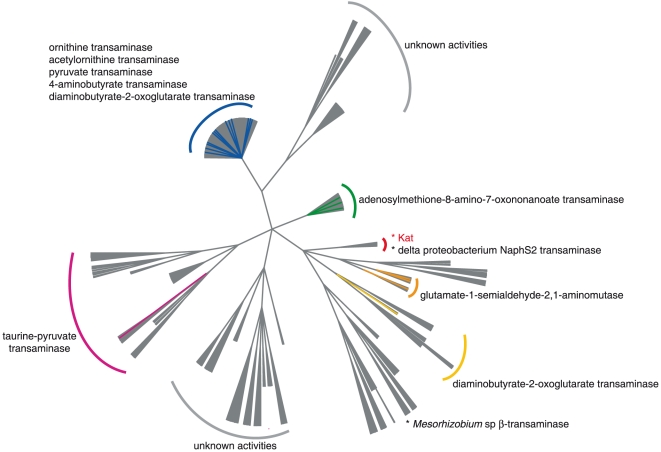
ASMC tree based on sequence and structural similarities of modelled active sites for 1343 enzymes belonging to the aminotransferases class III family (see Material and Methods). Known enzymatic activities are reported. Green: Adenosylmethionine-8-amino-7-oxononanoate transaminase. Red: 3-aminobutyryl-CoA transaminase. Orange: glutamate-1-semialdehyde-2, 1-aminomutase. Yellow: diaminobutyrate-2-oxoglutarate transaminase. Pink: taurine-pyruvate aminotransferase. Blue: ornithine, acetylornithine, pyruvate, 4-aminobutyrate, and diaminobutyrate-2-oxoglutarate transaminases.

On the basis of sequence similarity, aminotransferases can be grouped into subfamilies (class I to V). Kat is proposed as a class III aminotransferase since it contains a Pfam-A domain (PF00202). As the amino acid similarity between all the aminotransferases is low (∼30% identity), homology modelling appears to be a method of choice to compare protein sequences [31]. We have decided to build an aminotransferase tree including Kat from “*Candidatus* Cloacamonas acidaminovorans” based on a classification of active site homology models using the Active Sites Modelling and Clustering (ASMC) method [32]. ASMC is a phylogeny independent approach which permits to divide a family into sub-families depending on the composition of the active sites of its members. Since Kat does not show sequence homology with enzymes belonging to the other classes, the analysis was restricted to the class III aminotranferase family. A reduced set of 1343 sequences corresponding to a redundancy of 50% (UniRef50) was selected. Sequences from the digester that are homologous to Kat and the aminotransferases from NaphS2 and *Mesorhizobium* sp were added to this set. Results show that the sequence from “*Candidatus* Cloacamonas acidaminovorans” and the one from the reconstructed Bacteroidetes genome are clustered, beside other Kat homologous sequences from the anaerobic digester (figure 6). The aminotransferase from the delta proteobacterium NaphS2 belongs also to the same cluster. Ten additional enzymes have been classified in this cluster, but surprisingly, they are poorly related to Kat (∼30% identity) and belong to organisms not expected to ferment lysine (UniProtKB entry: A8ZXI1, D6ZIK6, A0YBF7, Q2GBS7, Q39NX5, D8F1V2, B5JCU4, C1A9A5, Q2J7L8, and C4YXY7). This suggests that structural features of the active site of Kat are shared by other members of the aminotransferases class III family. It is worthy to note that the enzyme from *Mesorhizobium* sp that metabolizes the same substrate as Kat (minus the CoA moiety) is located in a distinct cluster. One may hypothesize that in the Kat cluster, the enzymes are involved in acyl-CoA transamination.

### Conclusion

Taking advantage of the enormous metabolic potential of uncultivated microbial diversity, we have identified and kinetically characterized Kat, a novel β-transaminase involved in an alternative lysine fermentation pathway. The enzyme catalyzes the transamination of both aliphatic and aromatic substrates. β-aminoacids are chiral synthetic targets found in free forms, *i.e.* β-alanine or β-homoleucine, or as chiral moieties in molecules of pharmaceutical interest. Since β-transaminases catalyse the reductive amination of the prochiral keto group of β-ketoacids to produce optically pure β-amino acids, they could become useful tools for the organic chemist. Although several β-transaminases have been isolated to date, this enzyme is unique as it shows activity exclusively on thioesters (CoA and CoA mimics). This substrate specificity could be seen as an advantage because β-ketoacid substrates are prone to decarboxylation and are therefore difficult to manipulate. Coupling the enzymatic reductive amination catalyzed by this new β-transaminase with a thioester cleavage enzyme, would produce the β-aminoacids of interest.

The gene encoding this enzyme was detected using gene context methods on assembled sequences of a metagenome from the anaerobic digester of a wastewater treatment plant. To date, this gene seems to be found exclusively in this metagenome (with the exception of a gene from the the delta proteobacterium NaphS2, which obviously does not ferment lysine). Since *kat* is over represented as compared to *kal* (a distinctive gene of the classical pathway), one may suggest that a higher number of organisms ferment lysine according to the alternative pathway. It should be noticed that this pathway was found in organisms representative of two abundant phyla in the digester. This suggests that it should also be present in other organisms yet to be described. This raises the question of whether the classical pathway observed so far in cultivable organisms is actually representative. A complementary study of the occurrence of *kat* and *kal* in metagenomes from other digesters of wastewater treatment plants should address this issue, as representative sequences of the WWE1 lineage have been identified in at least two other anaerobic digesters [33]. More generally, this study raises a series of questions that will need to be answered, such as: is this kind of situation an exception or a common feature? And what is the biological relevance of the variant pathway?

## Materials and Methods

### Chemicals

All chemicals and enzymes were purchased from Sigma-Aldrich. Reagents for molecular biology were from Invitrogen. Oligonucleotides were from Sigma Genosys. Proteinase inhibitor Pefabloc SC was purchased from Roche Applied Science. The Lysonase™ Bioprocessing Reagent was from Novagen. (3*S*)-3-aminobutyryl-CoA was prepared by enzymatic conversion of 3,5-diaminohexanoate [13]. The compound was produced using 70 µg of 3,5-diaminohexanoate dehydrogenase, 140 µg of 3-keto-5-aminohexanoate cleavage enzyme, 30 mM 3,5-diaminohexanoate, 6 mM NAD^+^, and 4 mM acetyl-CoA in 500 µl 50 mM Tris/HCl pH 9.1. Once equilibrium was reached (as indicated by NAD^+^ reduction kinetics), the reaction was stopped by 1% 13 M trifluoroacetic acid. 3-aminobutyryl-CoA concentration was estimated by NADH formation. (*R,S*)-3-aminobutyryl-CoA was synthesized by Alpha Chimica from (*R*,*S*)-3-aminobutyrate. S-NAC thioesters were synthesized using conventional routes [34] (see Data S1).

### Construction of the expression vectors

The coding sequences of *hemL*-like (CLOAM0809) and atoA/atoD from “Candidatus *Cloacamonas acidaminovorans*” were amplified by PCR with the following primers:

CLOAM0809-fwd-5′-GCAGAAAAATTGAAATTAGCC-3′ and

CLOAM0809 -rev-5′-TTATAACATCTTTTTCACTTCG-3′;

*atoA*-fwd-5′-GCATTAGATAAACGAGCTATG-3′ and

*atoA*-rev-5′-TCAGAAGGTATTAACTCTTTC-3′;

*atoD*-fwd-5′-GTACAAATTATTAGTGCCGC-3′ and

*atoD*-rev-5′-TTAATTCTCCTTTGCTAAAAC-3′

The amplified sequences were inserted into the Invitrogen pEXP-5-NT/TOPO vector according to the manufacturer's protocol. The coding sequence of hemL-like (DIG1_60031) from the reconstructed genome affiliated with the Bacteroidetes phylum was amplified with the following primers:

DIG1_60031 -fwd-5′-AAAGAAGGAGATAGGATCATGCATCATCACCATCACCATTCAAAGAAACTAAAACTGGACG-3′ and

DIG1_60031 -rev-5′-GTGTAATGGATAGTGATCTTAAATATATTTTTTCAGTTCGCC-3′

The PCR product was inserted into the modified Novagen pET22b(+) vector (kindly provided by V. Döring) by directional cloning according to the ligation independent cloning method [35]. The sequence of the resulting plasmids, named pEXP5-HEML-LIKE, pEXP5-ATOA, pEXP5-ATOD, and pET22-HEML-LIKE, respectively, were verified.

### Expression and purification of the recombinant proteins

Cell culture, cell extracts, and protein purifications were conducted as previously reported [13].

### Analytical methods

Acetoacetyl-CoA formation was monitored at 310 nm, in the presence of Mg^2+^ ions, using a molar extinction coefficient of 11900 M^−1^.cm^−1^ at pH 8.1, as described by Stern *et al.* [36].

### Enzyme assays

Butyrate-acetoacetate CoA transferase activity was assayed in the presence of various substrates by monitoring the formation of acetoacetyl-CoA (acyl-CoA+acetoacetate↔acid+acetoacetyl-CoA). Reactions were performed in 100 µl of Activity Buffer (50 mM Tris/HCl pH 8.1) containing 10 mM acetoacetate, 15 µg of reconstituted AtoA/AtoD complex, and 4 mM MgCl_2_. The reaction was initiated by the addition of the acetyl-CoA. 3-aminobutyryl-CoA aminotransferase activity was assayed following acetoacetyl-CoA formation. Reactions were performed in 100 µl of Activity Buffer containing 5 mM MgCl_2_ in the presence of various concentrations of 3-aminobutyryl-CoA, α-ketoglutarate and pyruvate. The kinetic parameters were determined by varying one substrate concentration while keeping the other one at a fixed concentration. Kinetic constants were obtained from duplicate experiments by non-linear analysis of initial rates using SigmaPlot 9.0 (Systat Software, Inc.). Activity-pH and activity-temperature relationships were determined by incubating the enzyme at different pH values and constant temperature (28°C) and at constant pH (8.1) and different temperatures. As the apparent molecular extinction coefficient of acetoacetyl-CoA increases continuously with pH [37], its value was experimentally determined for each pH tested for enzyme activity. All enzymatic reactions were performed in a Safas UV mc^2^ or a Lambda 650 Perkin Elmer double beam spectrophotometer.

### LC/MS analyses

LC/MS analyses were carried out using a LTQ/Orbitrap mass spectrometer coupled to an Accela LC system (Thermo-Fisher). Chromatographic separation was conducted using an Acquity BeH C18 column (150×2 mm×1.7 µm; Waters) thermostated at 30°C. A mobile phase gradient was used with a flow rate of 0.4 ml/min in which mobile phase A consisted of 10 mM ammonium acetate adjusted to pH 4.0 with 0.1% (vol/vol) formic acid and mobile phase B consisted of methanol. The gradient started at 100% A for 1 min, followed by a linear gradient at 100% B for 7 min, and finally 5 min at 100% B. The entire eluant was sprayed into the mass spectrometer using a heated electrospray ionization source (175°C) at +4.5 kV with sheath, auxiliary and sweep gases set at 60, 45 and 8 arbitrary units, respectively. Desolvation of the droplets was further aided by setting the heated capillary temperature at 250°C. All metabolites were detected in the positive mode by full scan mass analysis from *m/z* 50–1000 at a resolving power of 30,000 at *m/z* = 400. Data dependant scanning was performed without use of a parent ion list. The resulting ion fragments were recorded in the LTQ linear trap.

### Sequence analysis

The genome sequence of “*Candidatus* Cloacamonas acidaminovorans” and the corresponding annotations were extracted from EMBL databank under accession number CU466930. The sequence of genes involved in the lysine fermentation pathway (FN1869, FN1868, FN1867, FN1866, FN1863, FN1862, *Fusobacterium nucleatum* ATCC 25586, AE009951) were compared to the “*Candidatus* Cloacamonas acidaminovorans” genome and to assembled genomic regions from the anaerobic digester using the BLAST2 algorithm [38]. These genes were also compared to “All Metagenomic Sequence Reads (N) » and All ORF Peptides available through the CAMERA web site (v1.3.2.29) [28] using the BLAST wizard algorithm. Putative orthologous relations between two genomes were defined as gene pairs satisfying the Bidirectional Best Hit criterion and an alignment threshold of 35% sequence identity over 80% of the length of the smallest protein. Synteny groups, *i.e.* conservation of the chromosomal co-localisation between pairs of orthologous genes from different genomes, were computed as previously described [39]. The data (*i.e.* syntactic and functional annotations, and results of comparative analysis) were stored in a relational database and explored by using the graphical interface of our microbial genome annotation system, MaGe. This database is publicly available at http://www.genoscope.cns.fr/agc/microscope/cloacamonascope [40]. Following the steps described in de Melo-Minardi *et al.* [32], homology models were built for each sequence and then aligned. Residues of catalytic pockets were extracted, and then submitted to a hierarchical clustering. The set of sequences for modelling was extracted from Uniprot (Pfam family PF00202; Aminotransferase class III). A reduced set of 1343 sequences corresponding to a redundancy to 50% (UniRef50) has been selected (http://www.uniprot.org/uniref/?query=uniprot%3a(PF00202)identity:0.5). Thirteen template structures were used for modelling the following enzymes: n-acetylornithine aminotransferase (PDB code: 2PB2, Chain: A), adenosylmethionine-8-amino-7-oxonanoate aminotransferase (1DTY, A), 7,8-diaminopelargonic acid synthase (1QJ5, A), 2,2-dialkylglycine decarboxylase (1D7R, A), gamma-aminobutyrate aminotransferase (1SFF, A), glutamate-1-semialdehyde aminotransferase (3K28, A), lysine aminotransferase (2CIN, A), omega-amino acid:pyruvate aminotransferase (3A8U, X), ornithine aminotransferase (1GBN, A), aminotransferase prk07036 (3I5T, A), d-phenylglycine aminotransferase (2CY8, A), alpha-amino-epsilon-caprolactam racemase (2ZUK, A), and aminotransferase class iii (3I4J, A). Half of these structures have been crystallized with a bound ligand in their active site.

## Supporting Information

Figure S1 Determination of the molecular weight of the AtoA/AtoD complex and Heml-like by Sephadex G-200 gel filtration. (•) molecular weight standards ovalbumin (mol. wt = 43,000), conalbumin (mol. weight = 75,000), aldolase (mol. wt = 158,000), ferritin (mol. weight = 440,000), and thyroglobulin (mol. weight = 669,000). (▵) Peak of eluted AtoA/AtoD complex. (○) Peak of eluted HemL-like.
(EPS)

Figure S2 Absorption spectrum of HemL-like recorded in Activity Buffer (50 mM Tris/HCl pH 8.1) after incubation with excess PLP and submitted to gel filtration.
(EPS)

Figure S3 pH- and temperature-dependent activity profiles of Kat from “*Candidatus* Cloacamonas acidaminovorans”. (*A*) pH-dependence of activity of Kat. Activities were measured at 28°C in the presence of 25 µM 3-aminobutyryl-CoA, 30 mM α-ketoglutarate, and 4 mM MgCl_2_. The buffers used (50 mM) were Tris/HCl (•), and glycine/NaOH (○). (*B*) Temperature-dependence of activity of Kat. Activities were determined in 50 mM Tris/HCl pH 8.1 with 25 µM 3-aminobutyryl-CoA and 30 mM α-ketoglutarate. Values correspond to the average of two replicates.
(EPS)

Table S1 Substrate specificity of AtoA/AtoD. Reactions were performed in 100 µl of Activity Buffer containing 4 mM MgCl2, 10 mM acetoacetate,15 µg of reconstituted AtoA/AtoD complex, and 100 µM acyl-CoA. 100% activity corresponds to 1.7 µmole of product/min/mg. Values correspond to the average of two replicates. ND: non detected.
(DOC)

Data S1 S-NAC thioesters synthesis.
(DOC)
