# Supplementary material for: A Novel Acyl-CoA Beta-Transaminase Characterized from a Metagenome
Source: PLoS One. 2011 Aug 3;6(8):e22918. doi: 10.1371/journal.pone.0022918 (PMC3149608; doi:10.1371/journal.pone.0022918)
Supplement: Table S1 — Substrate specificity of AtoA/AtoD. Reactions were performed in 100 µl of Activity Buffer containing 4 mM MgCl2, 10 mM acetoacetate,15 µg of reconstituted AtoA/AtoD complex, and 100 µM acyl-CoA. 100% activity corresponds to 1.7 µmole of product/min/mg. Values correspond to the average of two replicates. ND: non detected. (DOC) [file pone.0022918.s004.doc]

**Table S1**. Substrate specificity of AtoA/AtoD.

| Compound | Relative rate (%) |
| --- | --- |
| Butyryl-CoA  Acetyl-CoA  Malonyl-CoA  Crotonyl-CoA  Succinyl-CoA  3-aminobutyryl-CoA | 100  2.4  0.05  1.2  ND  ND |
