# Supplementary material for: A Novel Acyl-CoA Beta-Transaminase Characterized from a Metagenome
Source: PLoS One. 2011 Aug 3;6(8):e22918. doi: 10.1371/journal.pone.0022918 (PMC3149608; doi:10.1371/journal.pone.0022918)
Supplement: Data S1 — S-NAC thioesters synthesis. (DOC) [file pone.0022918.s005.doc]

**Data S1:** S-NAC thioesters synthesis.

NMR spectra were recorded on Spectrometers 300 MHz Brucker. Chemical shifts (expressed in ppm) of ^1^H and ^13^C NMR spectra were referenced to the solvent peaks ^1^H 3.31 and ^13^C 49.1 for CD_3_OD, ^1^H 2.50 and ^13^C 39.5 for DMSO-d6. MS analyses were carried out using a LTQ/Orbitrap mass spectrometer.

*Synthesis of S-NAC thioesters* ***10****,* ***11*** *and* ***12****:*

General procedure (1):

S-(2-acetamidoethyl)-3-aminobutanethioate (**10**)

To a DCM (17 mL) solution of 3-[(tert-butoxycarbonyl)amino]butanoic acid (**4**) (2) (1.32 mmol) at RT under N_2_, were added DMAP (0.2 eq.) and DCC (1.4 eq.). The reaction mixture was cooled to 0 °C and *N*-acetylcysteamine (1.3 eq;) in 4 mL of DCM was added. The reaction mixture was warmed up to RT and stirred for 48h. The white precipitate (DCU) was filtered through Celite, the cake was washed with EtOAc, the organic layers were assembled and the solvent were removed under reduced pressure. Purification of the product by flash column chromatography on silica gel (DCM then isocratic DCM/acetone: 83/17) afforded S-(2-acetamidoethyl) 3-[(tert-butoxycarbonyl)amino]butanethioate (**7**) as a colourless oil in 27% yield. ^1^H NMR (300 MHz, CDCl_3_) δ_1H_ 6.55 ( br s, 1H, N*H*-Boc), 4.73 ( br s, 1H, N*H*-C9), 4.08 (br s, 1H, H-2), 3.28 and 3.54 (m, 2H, H-7), 3.01 (m, 2H, H-6), 2.67 (m, 2H, H-3), 2.00 (s, 3H, H-10), 1.40 (s, 9H, (CH_3_)_3_ ), 1.19 (d, *J* = 6.9 Hz, H-1); ^13^C NMR (75 MHz, CDCl_3_) δ_13C_ 197.7 (C-4), 51.0 and 45.1 (C-2 and C-3), 39.3 (C-7), 30.6 (C-6), 28.6 (CH_3_ Boc), 22.8 (C-1), 21.1 (C-10).

TFA (2 mL) was added to a DCM (1 mL) solution of compound **7** (0.3 mmol). The reaction mixture was stirred at RT for 24h and the solvent were removed under vacuum to yield the TFA salt of S-(2-acetamidoethyl)-3-aminobutanethioate (**10**) in quantitative yield as a pale yellow oil. ^1^H NMR (300 MHz, DMSO-d6) δ_1H_ 8.09 (t, *J* = 5.3 Hz, 1H, N*H*-C9), 7.93 ( br s, 2H, NH_2_), 3.57 (m, 1H, H-2), 3.19 (m, 2H, H-7), 2.81-2.97 (m, 4H, H-3 and H-6), 1.78 (s, 3H, H-10), 1.19 (d, *J* = 6.6 Hz, 3H, H-1); ^13^C NMR (75 MHz, DMSO-d6) δ_13C_ 195.9 (C-4), 169.4 (C-9), 47.3 (C-3), 43.8 (C-2), 37.9 (C-7), 28.4 (C-6), 22.6 (C-10), 18.1 (C-1); HRMS (ESI+) calculated for C_8_H_17_N_2_O_2_S [M+H]^+^: 205.1011, found 205.0999.

S-(2-acetamidoethyl) 3-amino-5-methylhexanethioate (**11**)

S-(2-acetamidoethyl) 3-((tert-butoxycarbonyl)amino)-5-methylhexanethioate (**8**) was prepared from 3-((tert-butoxycarbonyl)amino)-5-methylhexanoic acid (**5**) (3). Purification by flash column chromatography on silica gel (petroleum ether/AcOEt: 1/9) afforded (**8**) in 74% yield as a white powder. ^1^H NMR (300 MHz, CDCl_3_) δ_1H_  6.46 ( br s, 1H, N*H*-C11), 4.63 (m, 1H, N*H*-Boc), 4.02 ( m, 1H, H-4), 3.55 (m, 1H, H-9), 3.24 (m, 1H, H-9), 3.02 (m, 2H, H-8), 2.57 (m, 2H, H-5), 1.98 (s, 3H, H-12), 1.63 (m, 1H, H-2), 1.39 (m, 9H, (CH_3_)_3_), 1.28 (m, 2H, H-3), 0.91 and 0.87 (d, 6.8Hz, 6H, H-1); ^13^C NMR (75 MHz, CDCl_3_) δ_13C_ 197.97 (C-6); 170.72 (C-11); 155.66 (C=O Boc); 79.66 (C-(CH_3_)_3_); 50.08 (C-5); 47.52 (C-4); 44.30 (C-3); 38.93 (C-9); 29.19 (C-8); 28.55 (CH_3_ Boc); 25.11 (C-2); 23.35 (C-12); 23.05 (C-1); 22.17 (C-1).

Boc cleavage afforded the TFA salt of S-(2-acetamidoethyl) 3-amino-5-methylhexanethioate (**11**) in quantitative yield as pale yellow oil. ^1^H NMR (300 MHz, CD_3_OD) δ_1H_  3.67 (m, 1H, H-4), 3.38 (m, 2H, H-9), 3.07 (m, 2H, H-8), 3.07-2.88 (m, 2H, H-5), 1.93 (s, 3H, H-12), 1.72 (m, 1H, H-2), 1.51 (m, 2H, H-3), 0.96 (m, 6H, H-1); ^13^C NMR (75 MHz, CD_3_OD) δ_13C_ 198.02 (C-6) ; 173.72 (C-11) ; 48.17 (C-5) ; 46.55 (C-4) ; 42.81 (C-3) ; 39.75 (C-9) ; 29.91 (C-8) ; 25.56 (C-2) ; 22.73 (C-12) ; 22.61 (C-1); HRMS (ESI+) calculated for C_11_H_23_N_2_O_2_S [M+H]^+^: 247.1475, found 247.1459.

S-(2-acetamidoethyl) 3-amino-3-phenylpropanethioate (**12**)

S-(2-acetamidoethyl) 3-((tert-butoxycarbonyl)amino)-3-phenylpropanethioate (**9**) was prepared from 3-((tert-butoxycarbonyl)amino)-3-phenylpropanoic acid (**6**) (4). Purification by flash column chromatography on silica gel (petroleum ether/AcOEt: 4/6 to 1/9) afforded (**9**) in 56% yield as a white powder. ^1^H NMR (300 MHz, CDCl_3_) δ_1H_  7.35-7.25 (m, 5H, H arom), 6.16 (br s, 1H, N*H*-C8), 5.24 (m, 1H, N*H*-Boc), 5.13 (m, 1H, H-1), 3.41-3.24 (m, 2H, H-6), 3.03 (m, 2H, H-5), 2.97 (m, 2H, H-2), 1.92 (s, 3H, H-9), 1.39 (s, 9H, (CH_3_)_3_); ^13^C NMR (75 MHz, CDCl_3_) δ_13C_ 211.74 (C-3), 158.53 (C-8), 135.54 (Cq arom), 129.00-126.43 (C arom), 78.12 (C-(CH_3_)_3_), 52.67 (C-2), 50.38 (C-1), 39.13 (C-6), 29.16 (C-5), 28.54 (CH_3_ Boc), 23.21 (C-9).

Boc cleavage and purification by flash column chromatography on silica gel (DCM/MeOH: 95/5 HCO_2_H 0.5%) yielded the formate salt of S-(2-acetamidoethyl) 3-amino-3-phenylpropanethioate (**12**) in 84% yield as a pale yellow oil. ^1^H NMR (300 MHz, CD_3_OD) δ_1H_  7.45 (m, 5H, H arom), 4.78 (m, 1H, H-1), 3.36-3.28 (m, 4H, H-2 and H-6), 3.02 (m, 2H, H-5), 1.90 (s, 3H, H-9); ^13^C NMR (75 MHz, CD_3_OD) δ_13C_ 197.17 (C-3), 173.67 (C-8), 137.74 (Cq arom), 130.64-130.50-128.44 (C arom), 53.26 (C-2), 53.18 (C-1), 39.82 (C-6), 29.72 (C-5), 22.67 (C-9); HRMS (ESI+) calculated for C_13_H_19_N_2_O_2_S [M+H]^+^: 267.1162, found 267.1148.

**Supplemental references.**

1. Brobst, S. W., and Townsend, C. A. (1994) *Canadian Journal of Chemistry-Revue Canadienne De Chimie* **72**, 200-207

2. McKiernan, M., Huck, J., Fehrentz, J. A., Roumestant, M. L., Viallefont, P., and Martinez, J. (2001) *Journal of Organic Chemistry* **66**, 6541-6544

3. Elmarini, A., Roumestant, M. L., Viallefont, P., Razafindramboa, D., Bonato, M., and Follet, M. (1992) *Synthesis-Stuttgart*, 1104-1108

4. Kumar, V., Jaggi, M., Singh, A. T., Madaan, A., Sanna, V., Singh, P., Sharma, P. K., Irchhaiya, R., and Burman, A. C. (2009) *European Journal of Medicinal Chemistry* **44**, 3356-3362
